# Altered Expression Profile of IgLON Family of Neural Cell Adhesion Molecules in the Dorsolateral Prefrontal Cortex of Schizophrenic Patients

**DOI:** 10.3389/fnmol.2018.00008

**Published:** 2018-01-29

**Authors:** Karina Karis, Kattri-Liis Eskla, Maria Kaare, Karin Täht, Jana Tuusov, Tanel Visnapuu, Jürgen Innos, Mohan Jayaram, Tõnis Timmusk, Cynthia S. Weickert, Marika Väli, Eero Vasar, Mari-Anne Philips

**Affiliations:** ^1^Department of Physiology, Institute of Biomedicine and Translational Medicine, University of Tartu, Tartu, Estonia; ^2^Centre of Excellence for Genomics and Translational Medicine, University of Tartu, Tartu, Estonia; ^3^Institute of Psychology, University of Tartu, Tartu, Estonia; ^4^Department of Pathological Anatomy and Forensic Medicine, University of Tartu, Tartu, Estonia; ^5^Estonian Forensic Science Institute, Tallinn, Estonia; ^6^Department of Chemistry and Biotechnology, Tallinn University of Technology, Tallinn, Estonia; ^7^Faculty of Medicine, School of Psychiatry, University of New South Wales, Sydney, NSW, Australia; ^8^Schizophrenia Research Institute, Neuroscience Research Australia, Randwick, NSW, Australia

## Abstract

Neural adhesion proteins are crucial in the development and maintenance of functional neural connectivity. Growing evidence suggests that the IgLON family of neural adhesion molecules LSAMP, NTM, NEGR1, and OPCML are important candidates in forming the susceptibility to schizophrenia (SCZ). IgLON proteins have been shown to be involved in neurite outgrowth, synaptic plasticity and neuronal connectivity, all of which have been shown to be altered in the brains of patients with the diagnosis of schizophrenia. Here we optimized custom 5′-isoform-specific TaqMan gene-expression analysis for the transcripts of human IgLON genes to study the expression of IgLONs in the dorsolateral prefrontal cortex (DLPFC) of schizophrenic patients (*n* = 36) and control subjects (*n* = 36). Uniform 5′-region and a single promoter was confirmed for the human *NEGR1* gene by *in silico* analysis. IgLON5, a recently described family member, was also included in the study. We detected significantly elevated levels of the *NEGR1* transcript (1.33-fold increase) and the *NTM* 1b isoform transcript (1.47-fold increase) in the DLPFC of schizophrenia patients compared to healthy controls. Consequent protein analysis performed in male subjects confirmed the increase in NEGR1 protein content both in patients with the paranoid subtype and in patients with other subtypes. In-group analysis of patients revealed that lower expression of certain IgLON transcripts, mostly *LSAMP* 1a and 1b, could be related with concurrent depressive endophenotype in schizophrenic patients. Additionally, our study cohort provides further evidence that cannabis use may be a relevant risk factor associated with suicidal behaviors in psychotic patients. In conclusion, we provide clinical evidence of increased expression levels of particular IgLON family members in the DLPFC of schizophrenic patients. We propose that alterations in the expression profile of IgLON neural adhesion molecules are associated with brain circuit disorganization in neuropsychiatric disorders, such as schizophrenia. In the light of previously published data, we suggest that increased level of NEGR1 in the frontal cortex may serve as molecular marker for a wider spectrum of psychiatric conditions.

## Introduction

Schizophrenia (SCZ) is a severe neuropsychiatric disorder with complex etiology, affecting approximately 1% of the world’s population (van Os and Kapur, 2009; Costain and Bassett, 2012). Schizophrenic patients have a reduced quality of life, significant functional impairment and suicide rates among schizophrenic patients remain alarmingly high (Pompili et al., 2007). High heritability of schizophrenia has been consistently demonstrated by family studies (Mäki et al., 2005) and genome-wide association studies have identified numerous risk chromosome loci candidates for schizophrenia (O’Donovan et al., 2008; Purcell et al., 2009; Stefansson et al., 2009; Ripke et al., 2013, 2014; Yue et al., 2017) indicating many common gene variants providing subtle effects (Giusti-Rodríguez and Sullivan, 2013). Evidence from GWAS and studies revealing differentially expressed proteins in schizophrenia suggests that the majority of the pathophysiological processes underlying schizophrenia are involved in neuronal transmission, synaptic maturation and plasticity, neurite outgrowth and neurogenesis (Hosak et al., 2012; Nascimento and Martins-de-Souza, 2015; Singh et al., 2017).

A growing body of evidence suggests the existence of certain genetically transmitted intermediate phenotypes for psychiatric disorders such as schizophrenia that determine the molecular setup of the neuronal substrate (Gottesman and Gould, 2003). Disrupted functional brain connectivity, reflecting abnormal integrity of neural tracts, has been suggested to be an endophenotype for schizophrenia (Li et al., 2017). Cell membrane adhesion molecules, such as the IgLON proteins, are crucial for the correct assembly of neural circuits. The IgLON superfamily of neural cell adhesion molecules are Gpi-anchored neural cell surface glycoproteins (Salzer et al., 1996) in the neural cell membrane, including Lsamp (limbic system associated membrane protein), NTM (neurotrimin), OPCML (opioid-binding cell adhesion molecule), Kilon/Negr1 (neuronal growth regulator 1) and IgLON5 (Vanaveski et al., 2017). IgLON proteins form homophilic and heterophilic complexes along the cell surface (Reed et al., 2004) and regulate neurite outgrowth (Akeel et al., 2011), dendritic arborization (Pischedda et al., 2014; Pischedda and Piccoli, 2016) and synapse formation (Hashimoto et al., 2009) both in the developing and adult brain.

Polymorphisms in the human IgLON genes have been linked with a variety of psychiatric conditions, including schizophrenia. Based on Gwas meta-analysis from the Psychiatry Genomics Consortium (Ripke et al., 2014), there is strongest evidence for the association of *OPCML* and *NEGR1* genes with schizophrenia, compared to other IgLONs. The Gwas meta-analysis data from Ripke et al. (2014) in the genomic regions of IgLONs has been visualized in Supplementary Figures S1–S3. In earlier studies, *OPCML* gene at 11q25 has been found to be a schizophrenia susceptibility locus in the European (O’Donovan et al., 2008) and Thai populations (Panichareon et al., 2012) and *NTM* has been significantly associated with schizophrenia in a genome-wide meta-analysis (Wang et al., 2010). Copy number variants in 11q25 where *NTM* and *OPCML* gene lie in close proximity have repeatedly been identified in patients with the diagnosis of schizophrenia (Magri et al., 2010; Ye et al., 2012). The most convincing evidence linking Lsamp with schizophrenia comes from protein studies in the post mortem brains: the level of the LSAMP protein is increased in the postmortem dorsolateral prefrontal cortex (DLPFC) (Behan et al., 2009) and in the synaptosome fraction of the orbitofrontal cortex (Velásquez et al., 2017) in patients with schizophrenia. Furthermore, Lsamp and NEGR1 proteins are significantly upregulated in the post-mortem anterior prefrontal cortex of the patients with schizophrenia compared to healthy controls (Cox et al., 2016). An association between schizophrenia and polymorphisms in the Lsamp gene has been shown in the European (Koido et al., 2014) and Chinese (Chen et al., 2017) subjects.

In our previous study (Vanaveski et al., 2017) we have shown a twin-promoter structure in *NTM* and *OPCML*, similar to the genomic organization reported for *LSAMP* (Pimenta and Levitt, 2004), characterized by two alternative promotors 1a and 1b, whereas mouse *Negr1* and *Iglon5* transcripts have a uniform 5′ region, suggesting a single promoter. As the human *NEGR1* gene has been expected to have putative alternative promoters in public databases, Ensembl^1^ and NCBI-aceview^2^, *in silico* analysis of the genomic structure of human *NEGR1* gene was performed before the experimental part of the current study for the optimal design of gene-expression assays.

The main focus of the study was to determine whether patients with schizophrenia express altered mRNA/protein levels of IgLON genes compared to controls in a well characterized cohort (Weickert et al., 2010). We examined the DLPFC (Brodmann area 46) as this region is important in the neuropathology of schizophrenia due to its role in executive cognitive functions (Harrison and Weinberger, 2005; Baumgartner et al., 2011; Catts et al., 2013). Alternative transcripts derived from 1a to 1b promoters of *LSAMP*, *NTM*, and *OPCML* were quantified separately by using a custom 5′-isoform-specific TaqMan gene-expression assay. As suicide is a major cause of death among patients with schizophrenia (Pompili et al., 2007), we also studied gene expression changes and other significant factors, such as substance abuse, in patients who committed suicide attempts or completed suicide compared with patients with no suicidal behaviors.

## Materials and Methods

### *In Silico* Analysis of Alternative First Exons of Human *NEGR1* Gene

Bioinformatic analysis of alternative first exons and respective upstream regions (5′UTR) of human *NEGR1* transcript was performed along with all ESTs present and with the *LSAMP* gene. All the alignments were performed with Clustal Omega Multiple sequence alignment service^3^. Alternative transcripts were further analyzed by mapping representative EST sequences to the *NEGR1* transcript and genome region. High quality ESTs spanning upstream of I or II exon were subsequently aligned to explore potential existence of alternative first exons. The available transcripts query was confirmed by BLAST search in NCBI Refseq and Non-Refseq databases. The putative transcriptional start site was analyzed using DBTSS database^4^. The sequences used in the current study were obtained from respective databases in Ensembl (release 89) or NCBI. The sequence identity numbers were obtained from Ensembl database and accession codes from NCBI RefSeq. The sequences for the multiple sequence alignment were procured from the NCBI site. The coding sequences in the FASTA format were used for the alignment. CLUSTALW multiple sequence alignment program was used for aligning the sequences for *OPCML*1a (NM_001012393.1), *OPCML*1b (NM_002545.3) *NTM* 1a (NM_001048209.1), *NTM* 1b (NM_016522.2), *LSAMP* 1a (AK299851.1), *LSAMP* 1b (NM_002338.3), *NEGR1* (NM_173808.2) and *IGLON5* (NM_001101372.2).

### Human Postmortem Brain Samples

Human DLPFC tissues were obtained from the New South Wales Brain Bank Network (Sydney, Australia). The complete cohort consisted of 37 schizophrenia (including 7 schizoaffective) postmortem brain samples and 37 controls with no history of psychiatric diagnosis. Schizophrenia subjects were diagnosed according to the Diagnostic and Statistical Manual of Mental Disorders IV (DSM-4). A detailed clinical and demographic characterization of the cohort has previously been described by Weickert et al. (2010): “Detailed cohort demographic information” can be found in the Supplementary Table S5 and “Clinical characteristics of schizophrenia cohort” can be found in the Supplementary Table S6 (Weickert et al., 2010). The summary of postmortem subject demography for control and schizophrenia groups can be find in Matosin et al. (2015, **Table 1**). The anatomical identification and dissection of the tissue and preparation of total RNA and total protein has been described in detail in Weickert et al. (2010). Antipsychotic drug treatment premortem was standardized to lifetime chlorpromazine equivalent for each patient. Antidepressant drug treatment history was also specified on a qualitative scale (yes/no). Sixteen patients had a history of drug abuse in the past, either cannabis (*n* = 10), amphetamines (*n* = 4), opiates (*n* = 3), LSD (*n* = 5) or undefined hallucinogen (*n* = 1). Many of the patients had multi-drug abuse history. The use of the New South Wales Brain Bank Network cohort was approved by the Human Research Ethics Committees at the University of Wollongong (HE99/222) and the University of New South Wales (HREC07261). For the inter-hemisphere analysis, brain samples were collected from 7 deceased subjects with no evidence of neuropathology and psychiatric diseases by the Department of Pathological Anatomy and Forensic Medicine, University of Tartu, Estonia and the Estonian Forensic Science Institute, Tallinn, Estonia. The DLPFC was bilaterally dissected from the rostro-caudal middle part of the middle frontal gyrus (Brodmann area 46 and lateral part of area 9) by qualified pathologists under the full ethical approval from the Research Ethics Committee of the University of Tartu (Approval no 223/T-4 from human research ethics committee).

**Table 1:** 1a/1b isoform specific primers for human IgLON gene transcripts optimized for the current study.

Gene		Forward primer	Probe sequence	Reverse primer
*LSAMP*	1a	5′-TCCTGTCCCTCTTCTCATTGC-3′	5′-AACCGAGGCACGGACAAC-3′	5′-TTCTTGTCTTCTACAACGCACCTG-3′
	1b	5′-GATTGCTCTGCCTTCTTCCCAC-3′		
*NTM*	1a	5′-TGGCTGCTCTGTGTCTCTTCC-3′	5′-CGGAGATGCCACCTT-3′	5′-GGTGACCCGGTTGTCAATAGTG-3′
	1b	5′-TCTCAGGCTGCTGTTCCTTGTA-3′		
*OPCML*	1a	5′-TCGGCGACAACTGCCCTGCT-3′		5′-GGTTACCCGGTCATCTATGGTA-3′
	1b	5′-TCTCAGGCTGCTGTTCCTTGTA-3′		
*NEGR1*		5′-TGCTCGAACCAGTGGCTGGC-3′	5′-TGGGCGGCCGTGGACAA-3′	5′-CCCTTTGAAGCTCCATCTTCCA-3′
*IgLON5*		5′-TGGCCGTCATCAGCCGAG-3′	5′-ACGCCACCCTCAGCTGCT-3′	5′-TGGAGCGGTTCAGCCAGGC-3′
*HPRT1*		5′-GACTTTGCTTTCCTTGGTCAGG-3′	5′-TTTCACCAGCAAGCTTGCGACCTTGA-3′	5′-AGTCTGGCTTATATCCAACACTTCG-3′

*5′ FAM-3′ BHQ probe was used for the detection of IgLON transcripts and 5′ VIC-3′ TAMRA probe was used for the housekeeping gene HPRT1*.

### Quantitative Real-Time Polymerase Chain Reaction (qRT-PCR) Analysis

**FIGURE 1:**
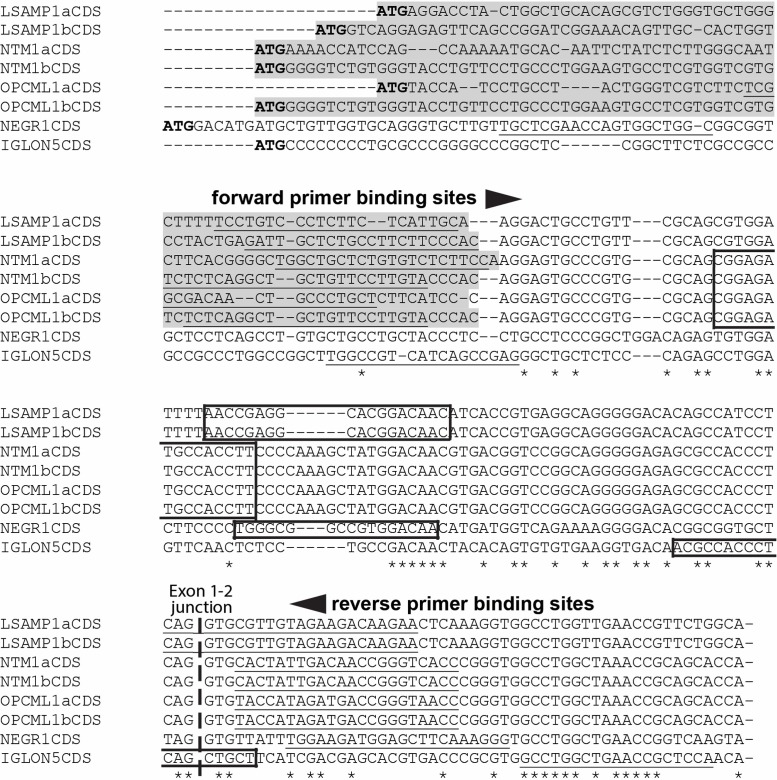
Multiple sequence alignment of the 5′ coding regions (CDS) of human IgLON gene family. The unique 5′-regions that are distinct in 1a/1b isoforms have been highlighted with gray shading. The binding sequences for FAM-BHQplus probes have been surrounded by a box. The binding sequences for 1a/1b isoform specific primers for human IgLON gene transcripts, have been underlined. The NCBI accession numbers of the human IgLON transcripts used as an input for CLUSTALW: *OPCML*1a (NM_001012393.1), *OPCML*1b (NM_002545.3) *NTM* 1a (NM_001048209.1), *NTM* 1b (NM_016522.2), *LSAMP* 1a (AK299851.1), *LSAMP* 1b (NM_002338.3), *NEGR1* (NM_173808.2) and *IGLON5* (NM_001101372.2). An ^∗^ (asterisk) indicates positions which have a single, fully conserved nucleotide.

IgLON transcript levels were determined by two-step RT-qPCR (qPCR). First strand cDNA was synthesized by the use of Random Hexamer (Applied Biosystems) and SuperScript^TM^ III Reverse Transcriptase (Invitrogen) according to the manufacturer’s protocol. Quantitative TaqMan Assay with FAM-BHQ-probe was designed for the detection of human *LSAMP* 1a/1b, *OPCML* 1a/1b, *NTM* 1a/1b, *NEGR1*, and *IGLON5* transcripts (see **Table 1** for the primer and probe sequences). The general primer design schema was similar for the primer design for mouse IgLON transcripts described previously in Philips et al. (2015). In case of *OPCML, NTM*, and *LSAMP* universal reverse primer was combined with an alternative forward oligo specific for either 1a or 1b transcript. The primer sequences have been mapped on the 5′-region of the IgLON transcripts in **Figure 1**. The sequences for IgLON transcripts and housekeeping gene *HPRT1* have been listed in 5′–3′ orientation in **Table 1**. TaqMan Universal PCR Master Mix was used according to the manufacturer’s protocol as reaction buffer. Two micrograms of RNA was used in 20 μl end reaction for cDNA synthesis from brain tissues. Each reaction mix was divided out in 10 μl quadruplicates. ABI Prism 7900HT Sequence Detection System with ABI Prism 7900 SDS 2.4.2 software (Applied Biosystems) was used for qPCR detection. qPCR data has been presented on linear scale, in form of 2^-ΔΔCT^ (Livak and Schmittgen, 2001) where ΔCT is the difference in cycle threshold (CT) between the gene of interest (FAM) and the housekeeper gene (VIC). The use of internal control gene, human *HPRT1*, has been optimized and used in our previous studies (Philips et al., 2010).

### Western Blot

The protein extraction for New South Wales Brain Bank Network has been described in Matosin et al. (2015). For inter-hemisphere analysis, frozen tissues were sonicated in ice cold RIPA buffer (Thermo Fisher Scientific) supplemented with protease inhibitor (Life Technologies). Protein lysates were centrifuged for 10 min at 12,000 *g* at 4°C. Supernatant was collected and protein concentration was determined by BCA method (Pierce BCA Protein Assay Kit, Thermo Scientific). Samples were frozen at -80°C for long-term storage. NuPAGE Electrophoresis System (Life Technologies) components and equipment were used according to the manufacturer’s instructions. Equal amounts of protein (20 μg) were loaded into the lanes of polyacrylamide gels. The gels were electrophoresed, followed by transfer of the protein to a nitrocellulose membrane using the NuPAGE Electrophoresis System (Life Technologies). The membranes were then blocked with 3% dry milk in PBS and probed with primary antibodies overnight at 4°C. Next day, immunoblots were incubated with fluorescent conjugated secondary antibodies for 1 h at room temperature, followed by visualization using a LI-COR Odyssey CLx system (LI-COR Biotechnologies). Images were converted to grayscale and data was analyzed using Image Studio Lite v 3.1.4 (LI-COR Biotechnologies). MILLIPORE Re-Blot Plus Western Blot Strong Antibody Stripping Solution (1x) was used to strip and re-probe membranes.

Primary antibodies used: mouse anti-NEGR1 (1:1,000) (sc-393293, Santa Cruz), mouse anti-Ntm (1:1,000) (sc-390941, Santa Cruz), rabbit anti-GAPDH antibody (1:10,000) (247002, Synaptic Systems). Secondary antibodies used: goat anti-rabbit antibody (1:40,000) (35569, Jackson ImmunoResearch) and goat anti-mouse antibody (1:5,000) (A21057, Invitrogen). Relative intensities for NEGR1 and NTM were obtained after normalization to GAPDH. Normalized values were divided by the lowest density value, so the lowest density value will be 1. The values were then normalized to control protein.

### Statistical Analysis of Human Gene Expression Data

Statistical analyses were performed with environment for statistical computing R - 3.3.2 and IBM SPSS Statistics 23. Significance level was set to *p* < 0.05 and data are presented as mean ± standard error of the mean. For analyzing gene expression data, non-parametric tests were applied, because not all of the gene expression data sets were normally distributed according to the Shapiro Wilk’s normality test. Mann–Whitney *U* test was used for comparing gene expression levels between two sample groups such as diagnostic groups (patient/control), as well as gender (male/female) and hemisphere (left/right). Additionally, Mann–Whitney *U* test was used for comparing gene expression levels within patient groups in case of binary categorical variables that were not applicable in the control group, such as antidepressant history (yes/no), concurrent major depression (yes/no), history of suicide attempts (yes/no) and death as a result of completed suicide (yes/no). Kruskal–Wallis test was used for comparing gene expression in more than two subgroups (diagnosis with and without schizoaffective disease vs. controls, agonal state, cause of death). *Post hoc* Tamhane’s test was carried out in order to determine the difference of groups averages more specifically. Spearman’s correlations were used to determine whether sample characteristics (tissue pH and RIN, post mortem interval, brain weight and volume and age at death) were associated with gene expression measures. Additional measures of disease characteristics were correlated specifically with the schizophrenia group (lifetime chlorpromazine equivalent reflecting antipsychotic drug history, age of disease onset and duration of illness). Analysis of covariance (ANCOVA) for diagnostic effects on protein expression was performed, accounting for factors that were associated with gene transcript levels, as determined by the Spearman’s correlations. Analyses of covariance were carried out only for *NTM* 1b expression data, which was distributed close to normal distribution (absolute values of skewness and kurtosis were less than 1) and the parametric approach was justified. A Chi-square test of independence was calculated, comparing the frequency of drug abuse in the groups of patients who committed suicide attempts or completed suicide and patients who did not. Binary logistic regression analysis was performed to assess the contribution of different gene expression levels to the model predicting the likelihood of being a patient. ROC analysis was performed to estimate predictive accuracy of the logistic regression model.

## Results

### *In Silico* Analysis of Human *NEGR1* Gene and Protein

*In silico* analysis of the human *NEGR1* transcript with 5′UTR region and ESTs present in the database shows no evidence of twin promoter structure in *NEGR1*. Our analysis showed a single CpG island present in the human *NEGR1* gene corresponding to the 5′UTR region of annotated variants NM_173808 (ENST00000357731.9). Additional EST analysis confirmed uniform 5′UTR region indicating single promoter structure for the uniform 5′-region of human *NEGR1* transcript encoding for single N-terminal signal peptide. Multiple sequence alignment of the 5′-regions of human IgLON gene family has been presented in **Figure 1**.

### Gene Expression Analysis in Patients in Comparison with Healthy Controls

**FIGURE 2:**
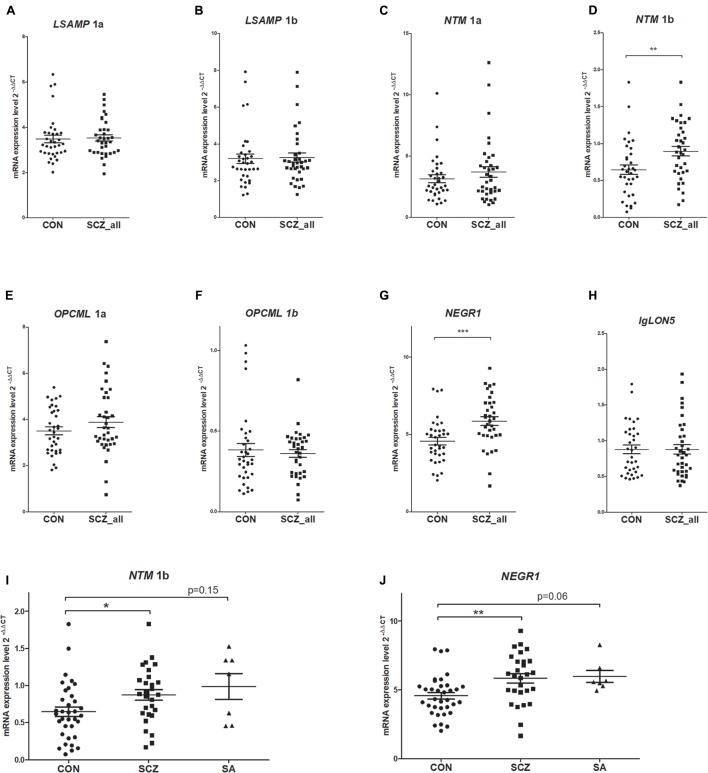
Expression levels in the brains of controls (CON, *n* = 36) and patients with schizophrenia (SCZ_all, *n* = 36) for *LSAMP* 1a **(A)**, *LSAMP* 1b **(B)**, *NTM* 1a **(C)**, *NTM* 1b **(D)**, *OPCML* 1a **(E)**, *OPCML* 1b **(F)**, *NEGR1* **(G)**, and *IgLON5* **(H)** transcripts. The patients with schizoaffective subtype (SA, *n* = 7) of schizophrenia were separated from the patient group (SCZ, *n* = 29) for *NTM* 1b **(I)** and *NEGR1* **(J)**. Whiskers represent means ± SEM. ^∗∗∗^*p* < 0.001, ^∗∗^*p* < 0.01, ^∗^*p* < 0.05 vs. control. As there was no significant difference between the gene expression values of the schizoaffective group and other schizophrenic patients (*p* = 1), we have used the data for all patients (SCZ_all) for the consequent analysis (such as logistic regression and ANCOVA).

The qPCR data from two subjects (one from the patient group and another from the control group) was excluded from the analysis due to insufficient quality of the reactions, therefore data from 36 patients to 36 control subjects was included in the gene expression studies. There was no effect of gender or hemisphere on the expression levels of IgLON transcripts according to the Mann–Whitney *U* test. The average gene expression values of patients were greater than averages of controls for all genes (except for *IGLON5*, **Table 2** and **Figure 2**). Mann–Whitney *U* test revealed statistically significant differences between patient and control groups only for *NTM* 1b and *NEGR1*. *NTM* 1b was significantly increased in schizophrenia subjects (*m* = 0.94) compared to controls (*m* = 0.64) (*p* < 0.001) (**Figure 2D**), and *NEGR1* was also significantly increased in schizophrenia subjects (*m* = 6.07) compared to controls (*m* = 4.57) (*p* < 0.001) (**Figure 2G**).

**Table 2:** Averages and standard deviations of groups of patients and controls.

	Average (standard deviation)		*W*	*P*-value
	Patients (*n* = 36)	Controls (*n* = 36)		
*LSAMP* 1a	3.66 (1.10)	3.48 (1.00)	548	ns
*LSAMP* 1b	3.25 (1.47)	3.19 (1.55)	598	ns
*NTM* 1a	3.70 (2.59)	3.16 (1.86)	574	ns
*NTM* 1b	0.94 (0.47)	0.64 (0.39)	377	*p* < 0.01
*OPCML* 1a	3.89 (1.42)	3.50 (0.99)	522	ns
*OPCML* 1b	0.41 (0.30)	0.38 (0.24)	567	ns
*NEGR1*	6.07 (2.07)	4.57 (1.45)	320	*p* < 0.001
*IGLON5*	0.87 (0.41)	0.87 (0.35)	658	ns

*W statistic from Mann–Whitney test. ns: not significant (*p* > 0.05)*.

When the patients with schizoaffective subtype (*n* = 7) of schizophrenia were separated from the patient group (**Figure 2I**), the Kruskal–Wallis test revealed a statistically significant difference in average expression values of *NTM* 1b between the groups (*H*^2^ = 9.11, df = 2, *p* = 0.011). Tamhane’s *post hoc* comparisons indicated that the mean score of patients with schizophrenia (*m* = 0.93, *sd* = 0.48) was statistically higher (*p* < 0.05) than the mean score of controls (*m* = 0.65, *sd* = 0.39). Although the mean score of patients with schizoaffective diagnosis (*m* = 0.99, *sd* = 0.46) was also higher than the mean of control subjects, the expression values of *NTM* 1b in the schizoaffective group were significantly different from neither controls (*p* = 0.15) nor the group of patients without the schizoaffective diagnosis (*p* = 1). Similarly, the Kruskal–Wallis test revealed a statistically significant difference in *NEGR1* levels between the groups when the patients with a schizoaffective subtype were separated from the patient group (*H*^2^ = 12.09, df = 2, *p* = 0.002) (**Figure 2J**). *Post hoc* comparisons using Tamhane’s test indicated that the mean score of patients with schizophrenia (*m* = 6.09, *sd* = 2.24) was statistically higher (*p* < 0.01) than the mean score of controls (*m* = 4.57, *sd* = 1.47), but the mean score of patients with a schizoaffective diagnosis (*m* = 5.97, *sd* = 1.13) did not differ significantly from the mean scores of patients without schizoaffective schizophrenia (*p* = 1); the difference between schizoaffective patients and controls showed a strong tendency toward statistical significance (*p* = 0.06).

Non-parametric correlation coefficient was used to determine whether sample characteristics were associated with gene expression measures. In the complete study group, the RIN of the sample was correlated with *LSAMP* 1a (*r* = -0.40, *p* < 0.01), *LSAMP* 1b (*r* = -0.33, *p* < 0.01), and *NTM* 1a (*r* = -0.49, *p* < 0.01) mRNA expression levels. Additionally, in the complete study group, pH of the tissue sample correlated with *LSAMP* 1a (*r* = -0.50, *p* < 0.01); *LSAMP* 1b (*r* = -0.49, *p* < 0.01), *NTM* 1a (*r* = -0.43, *p* < 0.01) and *NTM* 1b (*r* = -0.26, *p* < 0.05) mRNA expression levels. The ANCOVA analysis, accounting for tissue pH that was found to be correlated with gene expression measures, revealed that a significant difference in *NTM* 1b average values (patients vs. controls) was maintained after covarying for pH in the prefrontal cortex. More specifically, the total ANCOVA model was statistically significant (*p* < 0.01, $\eta_{p}^{2}$ = 0.16), and the averages of *NTM* 1b in patients and controls differed significantly (*p* < 0.01); the value of tissue pH was also significant in the model (*p* < 0.04).

Logistic regression was performed in order to assess the contribution of variables to the model predicting the status of being a patient. The dichotomous variable of being or not being a patient was used as dependent variable (**Table 3**). According to the model, the log of the odds of being a schizophrenia patient was positively related to the expression levels of *NTM* 1b and *NEGR1* (in both cases *p* < 0.05). In other words, the higher the *NTM* 1b expression, the greater the odds for belonging to the patient group (3.65 times greater odds), and also, the higher the *NEGR1* expression level, the greater the odds of being a patient (1.61 times). We also computed size of the area under the ROC curve. It was 0.79 showing that predictive accuracy of our logistic regression model is good (ROC curve has been visualized on the Supplementary Figure S4).

**Table 3:** Results of logistic regression model.

		95% CI for odds ratio		
	*B*(*SE*)	Lower	Odds ratio	Upper
Constant	-3.49 (1.05)			
*NTM 1b*	1.29 (0.64)	1.13	3.65	14.38
*NEGR1*	0.49 (0.18)	1.16	1.61	2.38

### Analysis within Patient Group

**FIGURE 3:**
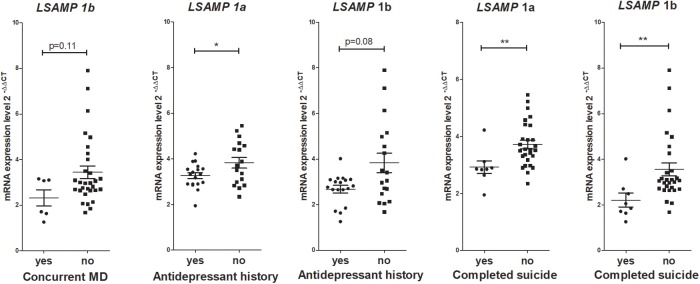
Gene expression analysis within patient groups revealed that lower expression of certain IgLON transcripts were associated with indicators of depression-related factors such as antidepressant treatment in the past or completed suicide. All six patients who had concurrent major depression (MD), also had antidepressant history in the past. At the same time, from the eight patients who committed suicide, only three had concurrent depression and history of antidepressant treatment. ^∗∗^*p* < 0.01, ^∗^*p* < 0.05 vs. control.

No significant correlations were found between gene expression values and measures of disease characteristics in the schizophrenia group, such as lifetime chlorpromazine equivalent reflecting antipsychotic drug history, age of disease onset and duration of illness. Analysis of gene expression levels within patient groups were performed in case of binary variables that were not applicable in the control group (**Figure 3**). Mann–Whitney *U* test revealed a significant difference in *LSAMP* 1a transcript expression levels in patients with antidepressant history (*n* = 18, *m* = 3.25, *sd* = 0.53) and patients with no antidepressant history (*n* = 18, *m* = 3.83, *sd* = 0.96); *w* = 109, *p* < 0.05; there were no other significant gene expression changes regarding antidepressant history of patients. The expression of *LSAMP* 1a transcript was significantly lower in patients who died as a result of completed suicide (*n* = 8, *m* = 2.92, *sd* = 0.63) than in patients who did not commit suicide (*n* = 28, *m* = 3.87, *sd* = 1.12) *w* = 189, *p* < 0.01. The expression of *LSAMP* 1b transcript was significantly lower in patients who committed a completed suicide (*n* = 8, *m* = 2.21, *sd* = 0.86) than in patients who did not commit suicide (*n* = 28, *m* = 3.55, *sd* = 1.48); *w* = 191, *p* < 0.05. The expression of *NTM* 1b transcript was significantly lower in patients who committed suicide (*n* = 8, *m* = 0.62, *sd* = 0.27) than in patients who did not commit suicide (*n* = 28, *m* = 0.97, *sd* = 0.48); *w* = 171, *p* < 0.05. The expression of *IgLON5* transcript was significantly lower in patients who committed suicide (*n* = 8, *m* = 0.56, *sd* = 0.12) than in patients who did not commit suicide (*n* = 28, *m* = 0.95, *sd* = 0.41); *w* = 181, *p* < 0.01; there were no other significant gene expression changes in patients who committed suicide compared with patients who did not commit suicide.

From the 37 patients in the current study cohort, 17 patients had a history of suicide attempts and 8 patients died due to completed suicide. Sixteen patients had a history of drug abuse in the past with cannabis being the most common drug used (*n* = 10). A Chi-square test of independence was calculated, comparing the frequency of drug abuse in the groups of patients who committed suicide attempts or completed suicide and who did not. A significant link was found between completed suicide and drug abuse when all drugs were included in the analysis: *c*^2^(1, *n* = 37) = 6.01, *p* < 0.05. Namely, the patients who committed suicide were more likely to have a drug abuse history (7 drug abusers from a total of 8 suicide cases) than patients who did not commit suicide (9 drug abusers from a total of 29 subjects). When only the use of cannabis was included in the analysis, again a significant association was found: *c*^2^(1, *n* = 37) = 9.01, *p* < 0.01. Namely, the patients who committed suicide were more likely to have a history of cannabis use (6 cannabis users from a total of 8 suicide cases) than patients who did not commit suicide (4 cannabis users from a total of 29 subjects). No significant association was found between suicide attempts and drug abuse when all drugs were included in the analysis: *c*^2^(1, *n* = 37) = 2.05, *p* = 0.15 (10 drug abusers from a total of 17 patients who committed suicide attempts vs. 6 drug abusers from 20 patients who had no history of suicide attempts). When only the use of cannabis was included in the analysis, a significant interaction was found: *c*^2^(1, *n* = 37) = 4.65, *p* < 0.05. Namely, the patients with a history of suicide attempts were more likely to have a history of cannabis use (8 cannabis users from a total of 17 attempters) than patients who did not commit suicide (2 users of cannabis from a total of 20 subjects). The amount of daily intake of ethanol showed no association with either suicidal behaviors or IgLON gene expression levels.

### Western Blot

**FIGURE 4:**
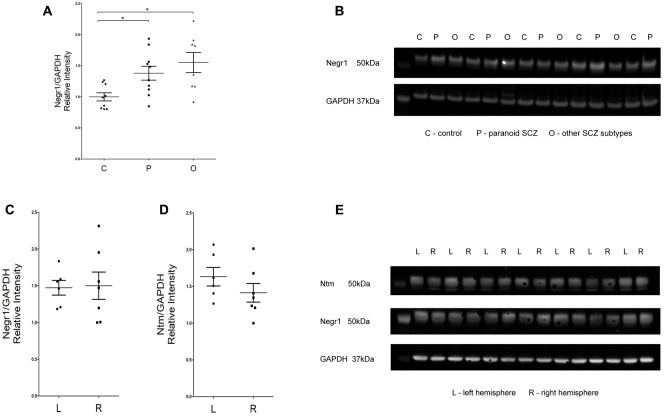
**(A,B)** Analysis of NEGR1 and representative immunoblots. DLPFC samples were collected from healthy (C, *n* = 9), paranoid SCZ (P, *n* = 10) and other SCZ subtype (O, *n* = 8) patients. **(C–E)** Analysis of NEGR1 and NTM in the left (L, *n* = 7) and right hemispheres (R, *n* = 7), and representative immunoblots. DLPFC samples were collected from healthy patients. Values are means ± SEM. ^∗^*p* < 0.05 vs. control.

According to the Kruskal–Wallis test, there was a statistically significant difference in NEGR1 protein level between the control group and the two subgroups of schizophrenia patients (*H*^2^ = 8.01, df = 2, *p* = 0.01) (**Figures 4A,B**). *Post hoc* comparisons using Tamhane’s test indicated that the mean NEGR1 protein expression score of schizophrenia patients with the paranoid subtype diagnosis (*m* = 1.72, *sd* = 0.43) was statistically higher than the mean score of controls (*m* = 1.24, *sd* = 0.24, *p* < 0.05). Additionally, the mean NEGR1 protein expression score of schizophrenia patients with another subtype diagnosis (*m* = 1.93, *sd* = 0.57) was statistically higher than the mean score of controls (*m* = 1.24, *sd* = 0.24, *p* < 0.05). As the hemispheres were randomly dissected in the complete study cohort, we performed inter-hemisphere analysis in the protein level by comparatively using left and right hemisphere-derived tissue samples in 7 subjects with no evidence of neuropathology and psychiatric diseases. Inter-hemisphere analysis revealed no obvious differences in protein expression; according to Mann–Whitney *U* test there were no significant differences in the NEGR1 and NTM proteins in the left and right hemisphere (**Figures 4C–E**).

## Discussion

Neural cell adhesion molecules, such as IgLON proteins, are critical in the formation of correct interactions between neural cells that underlie normal brain function and behaviors. Here we provide evidence of altered IgLON expression profiles in the DLPFC of patients with schizophrenia. We found that elevated levels of *NEGR1* transcript in the DLPFC increased the likelihood of being a patient 1.61 times; furthermore, increased levels of *NTM* 1b transcript isoform predicted the likelihood to fall into the patient group even 3.65 times. Consequent protein analysis performed in more distinct subgroups of male subjects confirmed the increased level of NEGR1 protein both in patients with the paranoid subtype and in patients with other subtypes. Currently the largest human post mortem gene expression analysis provides supporting evidence for the upregulation of *NEGR1* gene in the DLPFC area of schizophrenic patients (Fromer et al., 2016). However, this effect (*p* < 0.05) is not significant after multiple test correction. The data is still in line with our results, as whole transcriptome studies must apply stricter cut-off criteria to avoid false positive results. No evidence for differential expression of other IgLON genes, including *NTM* promoter specific isoforms, was provided by Fromer et al. (2016).

Interestingly, it has previously been shown that *NEGR1* transcript is significantly increased in the DLPFC of human subjects with major depressive disorder compared with non-psychiatric control subjects (Chang et al., 2014); another recent publication reports a strong association of polymorfisms in the *NEGR1* gene with major depression (Hyde et al., 2016). According to a genome-wide meta-analysis searching for common genetic variants influencing both schizophrenia and bipolar disorder, a polymorphism in the *NTM* gene (rs992564) accounts for the heritability of both disorders (Wang et al., 2010). A significant correlation between polygenic risk scores for bipolar disorder and the clinical dimension of mania in schizophrenia patients has been demonstrated recently (Ruderfer et al., 2014) expanding evidence that schizophrenia and bipolar disorder may share half of their genetic determinants (Wang et al., 2010). Elevated mean value of the *NEGR1* transcript in the DLPFC of schizoaffective patients did not meet statistical significance (**Figure 2J**) due to a small number of schizoaffective patients in the current study (*n* = 7), however, our results together with previous evidence (Chang et al., 2014; Cox et al., 2016) suggest that increased level of NEGR1 in the frontal cortex may serve as a molecular marker for a wider spectrum of psychiatric conditions.

Our previous findings underline the importance of differentiating 1a and 1b promoter activities when studying the IgLON family (Philips et al., 2015; Vanaveski et al., 2017). Uniform 5′ region of the human *NEGR1* gene (**Figure 1**) was confirmed by *in silico* analysis in the current study, suggesting a single promoter similarly to the genomic structure of the mouse *Negr1* gene (Vanaveski et al., 2017). The existence of putative alternative promoters predicted in public databases was not confirmed and the qPCR expression assay was designed based on human *NEGR1* transcript variant NM_173808.2, which is compatible with protein variant NP_776169.2.

Surprisingly, we found no changes in the levels of *LSAMP* transcripts, although LSAMP protein has been found to be increased in schizophrenic patients in the DLPFC (Behan et al., 2009), anterior prefrontal cortex (Cox et al., 2016), and also the synaptosome fraction of the orbitofrontal cortex (Velásquez et al., 2017) compared to healthy controls. An explanation could be that we measured separately alternative 1a and 1b promoter related transcripts of *LSAMP* that are not equivalent to the summarized level of protein in the brain. Furthermore, the presence of a conserved long 8 kb transcript of LSAMP has been shown (Pimenta et al., 1996), suggesting a variant with long poly-A tail which indicates that the translation levels of LSAMP may not be regulated only with the amount of mRNA. There are other studies showing that the level of mRNA might not always correspond to the level of protein, such as Matosin et al. (2015). At the same time, a significant increase of *NTM* 1b transcript in the DLPFC of schizophrenic patients indicates that distinguishing between alternative promoters is important and justified when studying the IgLON gene family. It is important to note that after cleavage of N-terminal 1a/1b-isoform specific signal peptides (encoded by 5′- coding region, **Figure 1**), the mature IgLON proteins are identical regardless of the initial promoter from which the expression of particular molecule was triggered (Pimenta and Levitt, 2004). Therefore, neither LSAMP nor NTM antibodies are able to discriminate between 1a and 1b isoforms that have highly different expressional distributions across the brain (Philips et al., 2015; Vanaveski et al., 2017) and supposedly differential roles. mRNA level analysis, such as qPCR, is the only possibility to separate promoter-specific isoforms in IgLONs.

The expression of *OPCML* uniform transcript has been explored in our cohort previously in a study which aimed to measure the expression levels of genes identified as schizophrenia susceptibility genes by GWASs (Umeda-Yano et al., 2014). No changes in the DLPFC of schizophrenic patients were found in the expression of *OPCML* uniform mRNA, similarly to our results, revealing no significant changes associated with schizophrenia in the expression of *OPCML* 1a and 1b isoforms. The work of Umeda-Yano et al. (2014) provides good evidence that genomic loci which have been detected as strong genetic determinants of a particular disease in GWASs might not necessarily give rise to expressional alterations in the closest gene, and important genomic regulatory areas may span over wider chromosomal region. Human *OPCML* and *NTM* genes lie approximately 68 kb apart on chromosomal locus 11q25, making it probable that these genes share regulatory mechanisms. One of the example is SNP rs11222692, located in the intronic area of the *NTM* gene, that is eQTL influencing the expression level of the *OPCML* gene in the DLPFC (Fromer et al., 2016). In our current study, we found significant alterations only in the *NTM* 1b transcript isoform. Alterations in 11q25 have already been associated with schizophrenia (Magri et al., 2010; Ye et al., 2012) and schizophrenia susceptibility has been associated with SNPs in both *OPCML* (O’Donovan et al., 2008; Panichareon et al., 2012) and *NTM* (Wang et al., 2010), making it a considerable risk locus for dysfunctional neural regulation (See also Supplementary Figure S3).

Post-psychotic depression, hopelessness, social isolation and a history of substance abuse, are the risk factors for suicide in patients with schizophrenia (Pompili et al., 2007). Interestingly, although the number of subjects was small in the current study, our study cohort provides support to the previously reported finding (Serafini et al., 2012) in that cannabis use may be a relevant risk factor associated with suicidal behaviors in psychotic patients. Further in-group analysis of patients revealed that lower expression of certain IgLON genes, mostly *LSAMP* transcripts, could be related with concurrent depression-related factors in schizophrenic patients (**Figure 4**). The expression of *LSAMP* 1b transcript was lower in patients with concurrent major depression than in patients with no major depression, but the difference was not significant due to a small number of patients with the diagnosis of concurrent major depression (*n* = 6). Both completed suicide and antidepressant episode can be interpreted as manifestations of the depressive endophenotype, however, in the current study we can only hypothesize that lower levels of IgLONs may be implicated in concurrent depressive traits and suicidality in schizophrenic patients as our study cohort is not including depressive patients and suicide victims without schizophrenia. Also, a notable shortcoming of the study is the significant correlation of *LSAMP* transcripts with the pH of the brain tissue, which may have an effect on the results. Still, the *LSAMP* gene has been associated with suicide susceptibility earlier (Must et al., 2008; Sokolowski et al., 2016), and therefore IgLONs, especially LSAMP, remain important targets in suicide genetics.

The current study has several limitations which have to be pointed out. As is common for post-mortem samples, there are many variables making the samples highly heterogeneous (such as tissue pH, brain weight and volume, RIN), which may have impact on the results. The heterogeneity of the cohort is further increased due to subjects’ individual history of drug abuse and the use of other psychoactive drugs, such as antidepressants. Additionally, our patient group was characterized by significant psychopathological differences which may also result in limited statistical power. Therefore especially the results derived from the subgroup analysis must be interpreted with caution.

We have shown alterations in the expression profile of IgLON transcripts in the frontal cortex of schizophrenic patients. IgLONs have been previously associated with different aspects of normal neural circuit formation. Specifically, NEGR1 has recently been shown to regulate neuronal arborization, playing a crucial role in the proper formation of neuronal connectivity (Pischedda et al., 2014; Pischedda and Piccoli, 2016). NTM has been shown to regulate the development of the thalamocortical and pontocerebellar projections (Struyk et al., 1995; Chen et al., 2001). Earlier studies have shown that the IgLON family functions collectively in a balanced manner by the formation of highly specific complexes and interactions between IgLON family members (Reed et al., 2004). We therefore propose that alterations in the expression profile of IgLON neural adhesion molecules are associated with brain network disorganization in neuropsychiatric disorders, such as schizophrenia.

## Conclusion

Our results provide clinical evidence beside numerous cell culture and animal experiments, confirming that certain members of the IgLON family of neural adhesion molecules, such as NEGR1, NTM, and LSAMP, are involved in fine-tuning of neuronal circuits underlying normal and pathological psychological/psychiatric conditions.

## Ethics Statement

This study was approved by the Human Research Ethics Committees at the University of Wollongong (HE99/222) and the University of New South Wales (HREC07261) (New South Wales Brain Bank Network cohort) and Research Ethics Committee of the University of Tartu (Approval no. 223/T-4 from human research ethics committee).

## Author Contributions

M-AP, KK, and EV planned the study. KK and MK prepared cDNAs and performed qPCR, K-LE performed Western Blot. KT and JI performed statistical analysis. MJ performed *in silico* analysis. CW, TT, TV, JT, and MV prepared and provided brain tissues, M-AP designed the qPCR assay. M-AP and KK wrote the paper. KK, MK, KT, JI, MJ, MV, TT, CW, and EV participated in data interpretation and revision of the paper and all authors approved the final version.

## Conflict of Interest Statement

The authors declare that the research was conducted in the absence of any commercial or financial relationships that could be construed as a potential conflict of interest. The reviewer ED and the handling Editor declared their shared affiliation.

## Footnotes

1 www.ensembl.org

2 https://www.ncbi.nlm.nih.gov

3 http://www.ebi.ac.uk/Tools/msa/clustalo/

4 http://dbtss.hgc.jp/
