# Supplementary material for: Altered Expression Profile of IgLON Family of Neural Cell Adhesion Molecules in the Dorsolateral Prefrontal Cortex of Schizophrenic Patients
Source: Front Mol Neurosci. 2018 Jan 29;11:8. doi: 10.3389/fnmol.2018.00008 (PMC5797424; doi:10.3389/fnmol.2018.00008)
Supplement: Supplementary file 1 [file Data_Sheet_1.PDF]

SUPPLEMENTARY MATERIAL

Altered expression profile of IgLON family of neural cell adhesion molecules in the dorsolateral prefrontal cortex of schizophrenic patients

Karina Karis[a,b], Kattri-Liis Eskla[a,b], Maria Kaare[a,b], Karin Täht[c], Jana Tuusov[d,e], Tanel Visnapuu[a,b], Jürgen Innos[a,b], Mohan Jayaram[a,b], Tõnis Timmusk[f], Cynthia Shannon Weickert[g,h], Marika Väli[d,e], Eero Vasar[a,b], Mari-Anne Philips[a,b]*

[a] Department of Physiology, Institute of Biomedicine and Translational Medicine, University of Tartu, 19 Ravila Street, 50411 Tartu, Estonia

[b] Centre of Excellence in Genomics and Translational Medicine, University of Tartu, Tartu, Estonia

[c] Institute of Psychology , University of Tartu , Tartu , Estonia

[d] Department of Pathological Anatomy and Forensic Medicine, University of Tartu, Tartu, Estonia

[e] Estonian Forensic Science Institute, Tallinn, Estonia

[f] Department of Chemistry and Biotechnology, Tallinn University of Technology, Tallinn, Estonia

[g] Faculty of Medicine, School of Psychiatry, University of New South Wales, Sydney, NSW, Australia.

[h] Schizophrenia Research Institute, at Neuroscience Research Australia, Randwick, NSW, Australia.

*** Correspondence:**
Dr. Mari-Anne Philips
marianne.philips@ut.ee

Supplementary figure S1. Human *NEGR1* gene in 1p31.1: R Graphics output based on the meta-analysis of GWAS of Schizophrenia (Ripke et al., 2014, https://data.broadinstitute.org/mpg/ricopili/; PGC_SCZ52_may13).

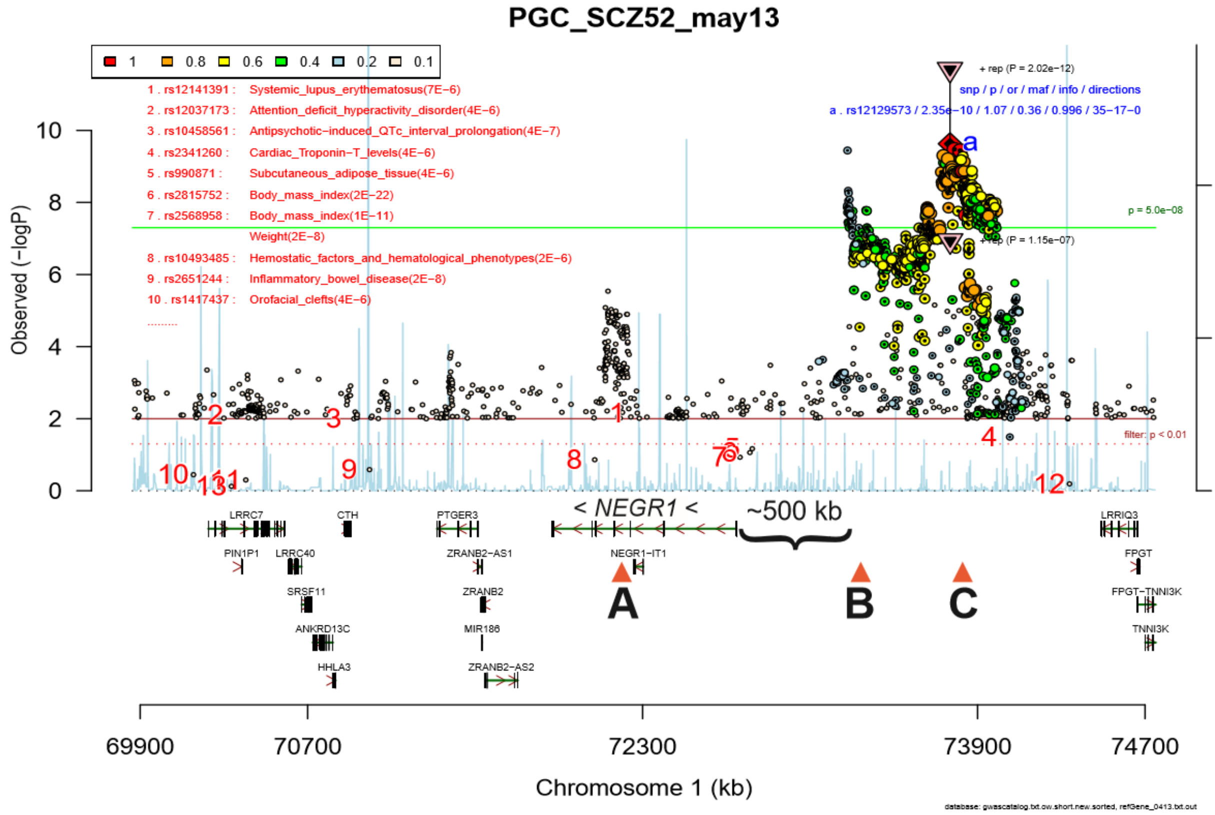

A. Based on the meta-analysis from Ripke et al. (2014) there is an association peak within the *NEGR1* gene, reaching a p-value around $10e^{-6}$ which does not exceed the genome-wide significance level ($p \leq 5 \times 10^{-8}$). **B.** However, there is a schizophrenia-associated locus ~500 kb upstream from the *NEGR1* gene, with a peak on rs35998080, which reaches the significance level with the value of $3.59e^{-10}$. **C.** The association locus that spans several hundreds of kb-s further upstream from the *NEGR1* gene reaches its highest peak at the significance level of $2.02e^{-12}$, corresponding to the locus rank 21 in the supplementary Table 3 of Ripke et al. (2014).

Supplementary figure S2. Human *NTM* and *OPCML* genes in 11q25: R Graphics output based on the meta-analysis of GWAS of Schizophrenia (Ripke et al., 2014, https://data.broadinstitute.org/mpg/ricopili/; PGC_SCZ52_may13).

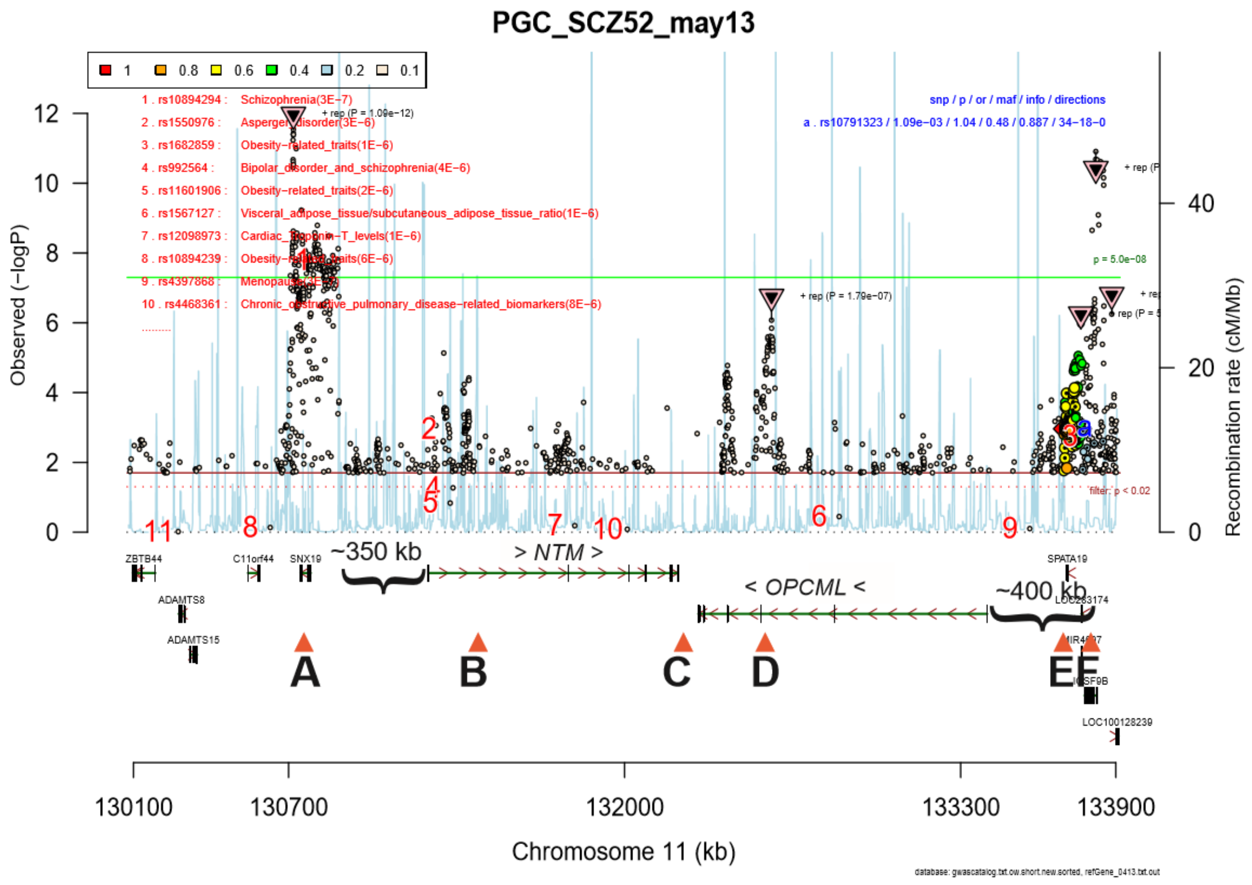

A. Based on the meta-analysis from Ripke et al. (2014) there is a schizophrenia-associated locus approximately 350 kb upstream from the *NTM* gene, exceeding the genome-wide significance level ($p \leq 5 \times 10^{-8}$), which corresponds to the locus rank 16 in the supplementary Table 3 of Ripke et al. (2014). However, the highest peak of the locus (p-value $1.09e^{-12}$), located ~500 kb upstream from *NTM*, is overlapping with another gene, *SNX19*, therefore *NTM* is not the primary candidate gene for this significant association. **B.** The approximate location of the SNP rs11222692 inside the intronic area of the *NTM* gene. According to Fromer et al. (2016), rs11222692 is eQTL influencing the expression level of the *OPCML* gene in the dorsolateral prefrontal cortex, indicating combined regulation of *NTM* and *OPCML*. **C.** *NTM* and *OPCML* lie approximately 68 kb apart. **D.** Based on Ripke et al. (2014), there is a schizophrenia-associated SNP rs2917569 in the intronic area of the *OPCML* gene, reaching the p-value of $1.79e^{-07}$. **E.** The genomic location of SNP rs10791323 has been shown, which is eQTL influencing the expression levels of the *OPCML* gene in the dorsolateral prefrontal cortex (Fromer et al., 2016). **F.** The significant schizophrenia associated locus ~400 kb upstream from *OPCML* reaches a significant p-value of $3.874e^{-11}$ and corresponds to the locus rank 36 in the supplementary Table 3 of Ripke et al. (2014). However, the peak is overlapping with another gene, *IGSF9B*, and therefore *OPCML* is not the primary candidate for this significant association. Hovever the location of eQTL rs10791323 for *OPCML* in the same region suggests that this region consists regulatory regions for the *OPCML* gene.

Supplementary figure S3. Human *LSAMP* gene in 3q13.31: R Graphics Output based on the meta-analysis of GWAS of Schizophrenia (Ripke et al., 2014, https://data.broadinstitute.org/mpg/ricopili/ PGC_SCZ52_may13).

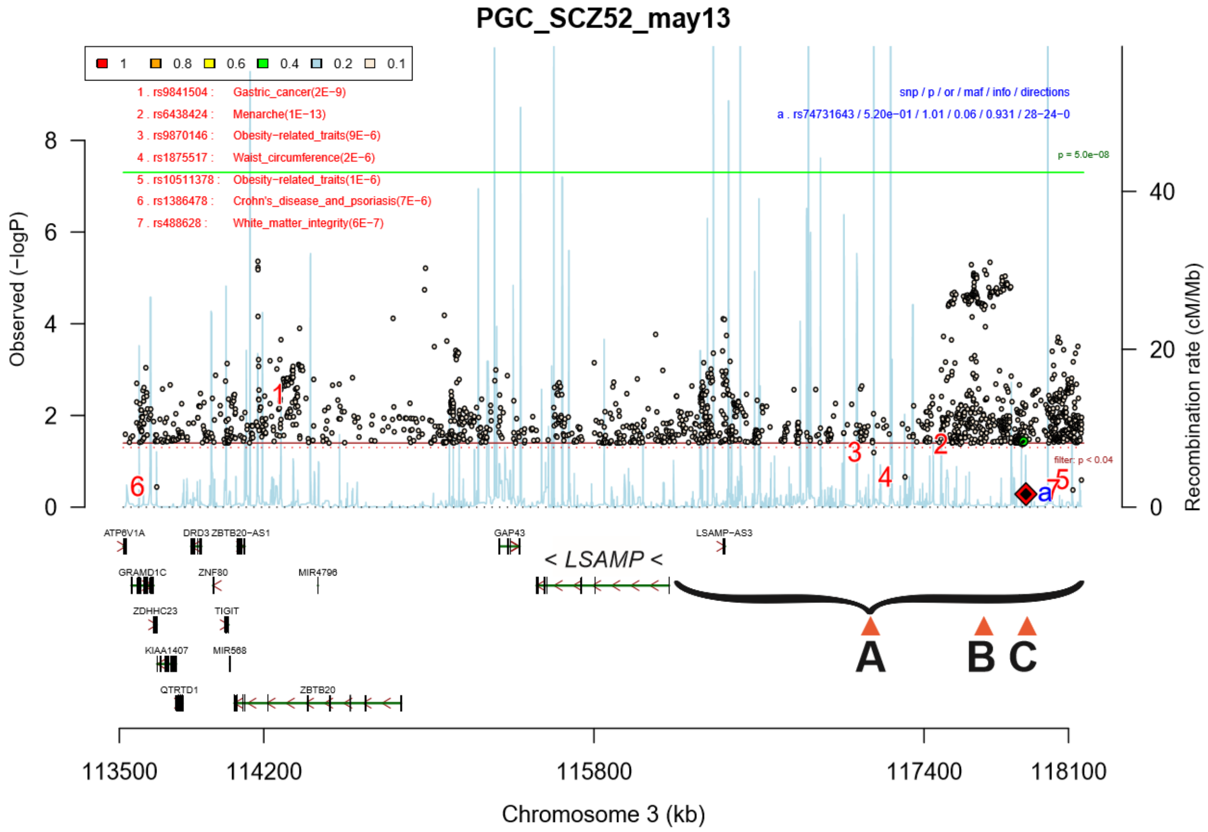

A. The genomic region upstream from the *LSAMP* gene has been described as a “desert gene” region extending 2.099 Mb (chr3: 116,520,120-118,619,479) (Gimelli et al., 2016). Based on the meta-analysis from Ripke et al. (2014) there are no schizophrenia-associated loci close to the *LSAMP* gene exceeding the genome-wide significance level ($p \leq 5 \times 10^{-8}$). **B.** The closest association peak lies ~1500 kb upstream from the *LSAMP* coding sequence reaching the p-value around $10e^{-6}$. There is evidence that the long “desert gene” region contains regulatory regions for the *LSAMP* gene. **C.** The approximate location of the SNP rs74731643 has been shown in the image, which is eQTL influencing the expression levels of the *LSAMP* gene in the dorsolateral prefrontal cortex (Fromer et al., 2016). The location rs74731643 has also been indicated as a red square with “a”.

Supplementary figure S4. Receiver Operating Characteristic (ROC) summarizes the model's performance by evaluating the trade offs between true positive rate (sensitivity) and false positive rate (1- specificity).

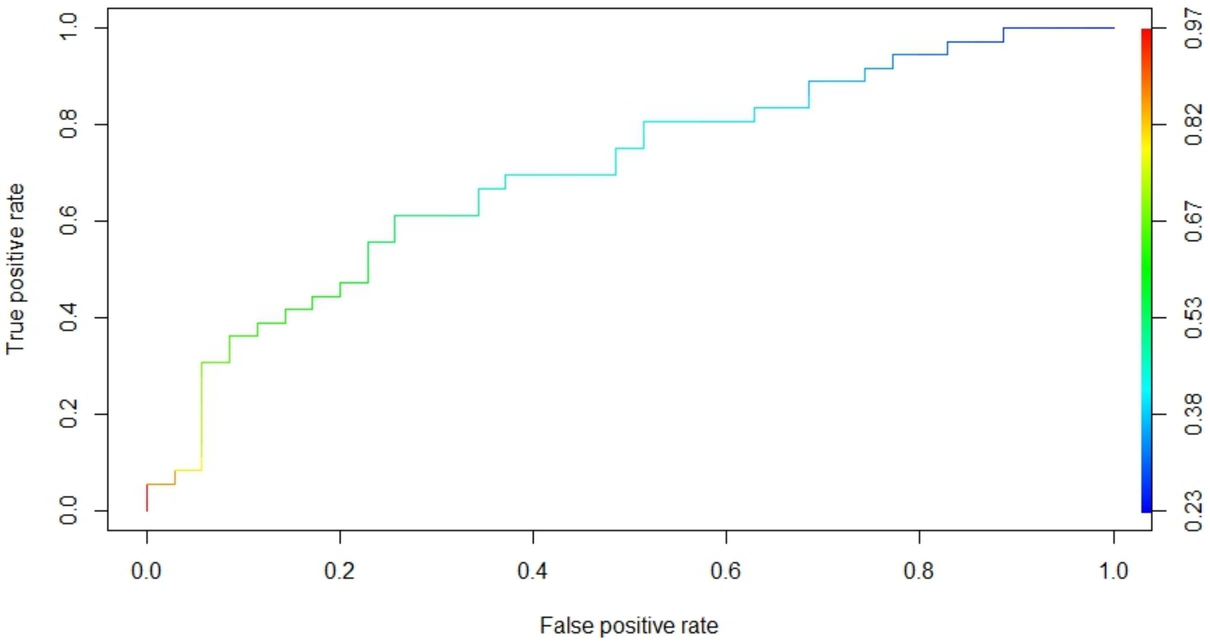

References for Supplement:

Fromer, M., Roussos, P., Sieberts, S. K., Johnson, J. S, Kavanagh, D. H., Perumal, T. M., et al. (2016). Gene expression elucidates functional impact of polygenic risk for schizophrenia. *Nat Neurosci* 19(11), 1442-1453. doi: 10.1038/nn.4399 https://www.synapse.org/#!Synapse:syn2759792/wiki/69613

Gimelli, S., Leoni, M., Di Rocco, M., Caridi, G., Porta, S., Cuoco, C., et al. (2013). A rare 3q13.31 microdeletion including *GAP43*and *LSAMP* genes. *Mol Cytogenet* 6(1):52. doi: 10.1186/1755-8166-6-52

Ripke, S., Neale, B. M., Corvin, A., Walters, J. T., Farh, K. H., Holmans, P. A., et al. (2014). Biological insights from 108 schizophrenia-associated genetic loci. *Nature* 511(7510), 421-427. doi: 10.1038/nature13595 https://data.broadinstitute.org/mpg/ricopili/ PGC_SCZ52_may13
